# Medical College of South Carolina

**Published:** 1860

**Authors:** 


### Medical College of South Carolina.

We have received the catalogue of students in the Medical College of South Carolina, for the session of 1859–60, from which we learn that their list of Matriculates amounted to 248, from the following States: South Carolina, 188; North Carolina, 10; Alabama, 23; Georgia 10; Florida, 6; Mississippi, 5; Virginia, 3; Connecticut, 1; Texas, 1; Kansas, 1.

We are greatly pleased to note the increasing prosperity of Southern Medical Colleges, furnishing as they do, all the facilities for a complete medical education, and among the number, we can commend the Medical College of South Carolina, as one of the most deserving, as it is one of the most prosperous.

